# Psychosocial factors influencing contraceptive use among adolescent mothers in the Volta Region of Ghana: application of the Health Belief Model

**DOI:** 10.4314/ahs.v21i4.43

**Published:** 2021-12

**Authors:** Paa Yeboah Akonor, Martin A Ayanore, Judith A Anaman-Torgbor, Elvis E Tarkang

**Affiliations:** 1 School of Public Health, University of Health and Allied Sciences PMB 31 Ho, Ghana; 2 School of Nursing and Midwifery, University of Health and Allied Sciences PMB 31 Ho, Ghana; 3 HIV/AIDS Prevention Research Network Cameroon PO Box 36 Kumba, Cameroon

**Keywords:** Contraceptive use, Adolescent mothers, Health Belief Model, Volta Region, Ghana

## Abstract

**Background:**

Globally, complications arising from pregnancy and childbirth are the leading cause of death among pregnant women aged 15 to 19 years. This study investigated the psychosocial factors influencing contraceptive use among adolescent mothers in the Volta Region using the Health Belief Model (HBM).

**Methods:**

This was a cross-sectional study conducted among 422 adolescent mothers. An interviewer-administered structured questionnaire was used to collect data. Binary logistic regression was used to determine the strength of association between the independent and the dependent variables at a p-value less than 0.05.

**Results:**

The prevalence of contraceptive use was 18.7%. Concerning perceived susceptibility, there was a significant association between contraceptive use and the perception of being at risk of pregnancy complications (p<0.001). Perceived barriers showed a significant association between fear of side-effects of contraceptives and contraceptive use (p=0.007). Concerning perceived self-efficacy, there was a significant association between contraceptive use and confidence to suggest to a partner to use contraceptives (p=0.003); confidence to ask for contraceptives from health facilities (p<0.001) and confidence to use contraceptives (p<0.001).

**Conclusion:**

Programmes to increase contraceptive use should aim at heightening the perception of risk of pregnancy complications, reduce barriers to contraceptive use and increase the skills in negotiating contraceptive use.

## Introduction

Contraceptive use as a method of family planning helps people to give birth to the number of children they desire as well as assist to determine the spacing of pregnancies^1^. Adolescent mothers between the ages of 10 and 19 years face a high risk of eclampsia, puerperal endometritis and systemic infections whiles babies born to them face a high risk of low birth weight, severe neonatal conditions and pre-term delivery^1^.

According to the World Health Organization (WHO) (2013), the infant mortality rate for children born to adolescent mothers is significantly higher than children born to non-adolescent mothers at 9.8 deaths per 1000 live births against 6.75 per 1000 live births respectively^2^. Globally, there is evidence showing that contraceptive use can help to prevent about 2.7 million infant deaths and the loss of 60 million healthy lives in a year^3^.

In Ghana, however, contraceptive use is low, which is a major public health concern. For instance, the current use of contraceptives is 23% among women of all ages^4^. The prevalence of contraceptive use among adolescents in the age group 15–19 years is the lowest (19%)^4^. According to Wilson, Ameme and Ilesanmi (2017)^5^, the age group 15–19 years, represents the highest regarding not using contraceptives in the Volta Region of Ghana. The 2014 Ghana Demographic and Health Survey (GDHS) also reported that 14% of women aged 15–19 years have begun childbearing (they have either had a live birth (11%) or are pregnant with their first child (3%))^4^. This is a slight increase from 13% in 2008^6^. The Volta Region is ranked first in terms of adolescent pregnancy with a rate of 22% in the country^4^.

The increased use of contraceptives among adolescent mothers can help to achieve two targets of the Sustainable Development Goal (SDG) 3: reducing the global maternal mortality ratio to less than 70 per 100,000 live births and ending preventable deaths of newborns and children under 5 years of age by the year 2030. It can also help to achieve SDG 5: gender equality and empower all women and girls^7^.

Every successful health promotion programme is based on theories about how and why people change their behaviours. The Health Belief Model (HBM) was developed to address this issue. This paper uses the main constructs of the HBM, namely perceived susceptibility, perceived severity, perceived benefit, perceived barriers and perceived self-efficacy, as the theoretical framework to determine the psychosocial factors influencing contraceptive use among adolescent mothers in the Volta Region of Ghana.

The HBM is built on the premise that:

- An adolescent mother will take a health-related action (contraceptive use) if she believes that a negative health condition can be avoided
- An adolescent mother will avoid a negative health condition if she has a positive expectation of the recommended action (contraceptive condom use)
- An adolescent mother will take a health-related action if she believes that she can successfully take the recommended health action (contraceptive use comfortably and with confidence)^8^,^9^.

The HBM has been applied to various health promotion behaviours such as osteoporosis prevention^10^,^11^; prevention of heart failure^12^–^14^; oral health^15^; obesity and overweight^16^; management of type 2 diabetes^17^; HIV risk perception^18^ and condom use^19^. However, there is paucity of studies that have applied the HBM to contraceptive use among adolescents; thus, justifying its use in the current study determine the psychosocial factors influencing contraceptive use among adolescent mothers in the Volta Region of Ghana.

Although adolescent mothers are part of the at-risk groups when it comes to maternal mortality, there has not been any study done in the Volta Region on the factors influencing contraceptive use among adolescent mothers grounded on a theory, even though the Region has a very high adolescent pregnancy rate.

## Method

### Study design

A cross-sectional study was conducted among 422 adolescent mothers in three Districts in the Volta region.

### Study site

Three Districts were selected namely North Dayi District, Hohoe Municipality and Ho Municipality. North Dayi District was selected because it had the highest percentage (20.3%) of adolescents (10–19) Antenatal Clinic (ANC) attendance in 2017. Hohoe Municipality was also selected because it had an average percentage of adolescents (10–19) ANC attendance in 2017 of 15%. Ho Municipality was selected because it had the lowest percentage (9.6%) of adolescents (10–19) ANC attendance in 2017, according to the information from the District Health Management Data^20^. This gave an unbiased representation of the Volta Region.

### Study population

The study population was adolescent mothers attending Postnatal Clinic (PNC) at 12 selected health facilities in North Dayi District, Hohoe Municipality and Ho Municipality.

### Sample size determination

The sample size for the study was calculated using the Magnani sampling guide^21^. Thus, the sample size required for the study was 384, calculated as follows:

N= Z2 × p(1-p)/d^2^

where N is sample size; z is reliability coefficient (1.96); d is margin of error (0.05); p is prevalence of contraceptive use among adolescent mothers (50%).

Therefore, N=1.962 × (0.50) (0.50)/0.052 =384

Adding a10% non-response rate of 38, gave an overall sample size of 422.

### Sampling procedure

The study employed a multistage sampling technique

#### Stage 1

Selection of Districts- Three Districts for the study were purposively selected if they fell in the high, middle and low clusters respectively. All the Districts were put into 3 clusters namely high cluster, middle cluster and low cluster regarding the 2017 Volta Regional adolescents (10–19 years) ANC attendance. Districts with a percentage between 20.3 and 16.4 were classified as a high cluster, those with a percentage between 15.5 and 14.5 were classified as middle cluster and those with a percentage between 14.3 and 9.6 were classified as a low cluster. North Dayi, Hohoe and Ho were purposively selected from the high, middle and low clusters respectively.

#### Stage 2

Selection of health facilities- In each of the Districts, all the names of the health facilities providing PNC services were written on pieces of paper, wrapped and placed in a container. Four persons not involved in the study were asked to pick one wrapped paper each without replacement from the container. Twelve facilities were thus randomly selected (4 from each District).

#### Stage 3

Selection of participants- Convenience sampling was used to identify and invite participants based on the calculated sample size. The purpose of the study was explained to the nurses by the research team. The nurses explained the purpose of the study to the adolescent mothers who came for PNC services and directed them to the study team. The purpose of the study was further explained to the mothers by the research team. Participant information and consent forms (PICFs) were given to the participants and the study team members were available to answer questions raised by the participants. The PICFs were explained to participants who could not read and verbal consent was obtained from this category of participants. Participants below 18 years were followed home to obtain parental consent while they gave their assent.

The sample size for the study was calculated using the Magnani sampling guide

### Instrument and data collection

A pretested structured interviewer-administered questionnaire was used for the data collection. The questionnaire was pretested among 20 adolescent mothers in the Cape Coast Metropolitan in the Central Region of Ghana. The pretesting aimed at assessing relevance, clarity, accuracy and flow of questions asked, the approximate time needed for each tool and the clarity of the instructions to the respondents. The refined questionnaires were administered to a sample of 422 adolescent mothers attending PNC in 12 health facilities (4 from each selected district) in the Volta Region. Research assistants who were fluent in English and Ewe languages were trained for the data collection.

The questionnaire contained three sections. Section A contained questions about demographic characteristics, Section B included questions on the utilization of contraceptives, and Section C contained the factors influencing contraceptive use based on the constructs of the HBM (perceived susceptibility, perceived severity, perceived benefits, perceived barriers, cues to action and perceived self-efficacy).

### Measures

#### Outcome (dependent) variable

contraceptive use Contraceptive use was measured with the following question: ‘Have you used any contraceptive before? The response options were ‘0=No’ (reference category)’ and ‘1=yes’.

### Independent variables: constructs of the HBM

Perceived susceptibility to adolescent pregnancy complications: This was measured based on the responses to the following questions each considered separately: ‘Do you know that if you do not use contraceptives you could be susceptible to adolescent pregnancy complications?’ and ‘Would you use contraceptives to prevent adolescent pregnancy complications?’ The response options were categorized into ‘0=No’ and ‘1=yes’. The reliability coefficient for perceived susceptibility items was 0.99.

Perceived severity of adolescent pregnancy complications: This measure was based on the responses to the following questions each considered separately: ‘Do you know that adolescent pregnancy-related complications can result in the death of the mother?’; ‘Do you know that adolescent pregnancy-related complications can result in the death of your baby?’; ‘Do you know that adolescent pregnancy-related complications can affect your reproductive system?’ and ‘Do you know that adolescent pregnancy-related complications can affect the child's brain?’ The response options were categorized into ‘0=No’ and ‘1=yes’. The reliability coefficient for perceived severity items was 0.98

#### The perceived benefit of using contraceptives

This measure was based on the responses to the following questions each considered separately: ‘Does contraceptive use prevent pregnancy effectively?’; ‘Does contraceptive use help in birth spacing?’ and ‘Does contraceptive use help one to achieve her goals like attaining higher education and getting good jobs?’ The response options were categorized into ‘0=No’ and ‘1=yes’. The reliability coefficient for the perceived benefit items was 0.97.

#### Perceived barriers to contraceptive use

This measure was based on the response to the following question: ‘What are some of the barriers that may prevent you from using contraceptives?’ with the following response options: distance to the acquisition of contraceptives, fear of side effects, costly, the attitude of service providers, partner or family opposition. The response for each option was categorized into ‘0=No’ and ‘1=yes’. The reliability coefficient for the perceived barrier was 0.61.

#### Cues to action for contraceptive use

This measure was based on the responses to the following questions each considered separately: ‘Do you get any information or reminders about contraceptives’? and ‘Have the information or reminders influenced you to use contraceptives’? The response options were categorized into ‘0=No’ and ‘1=yes’. The reliability coefficient for cues to action was 0.33.

#### Perceived self-efficacy for contraceptive use

This measure was based on the responses to the following questions each considered separately: ‘Do you have the confidence to suggest to your partner to use contraceptives?’; ‘Are you confident to ask for contraceptive methods in a health facility?’ and ‘Are you confident to use contraceptives?’ The response options were categorized into ‘0=No’ and ‘1=yes’. The reliability coefficient for self-efficacy was 0.85.

#### Socio-demographic variables

The following socio-demographic variables were included in the study: age, categorized into two groups (below 18 and 18 and above), marital status, (married and not married), religion (Christianity, Islam and Traditional), educational level, (none, primary, secondary and tertiary), parity level, (parity 1 and more than 1), ethnicity, (ewe, guans, and others), occupation, categorized into two groups (none and traders).

### Data analysis

Data collected from the study were entered into Epi Data 3.1 and exported into STATA 14.0 for cleaning and analysis. Binomial logistic regression was performed to examine the probability of using the female condom during sexual intercourse. Binomial logistic regression predicts the probability that an observation falls into one of two categories of a dichotomous dependent variable (female condom use) based on one or more independent variables that can be either continuous or categorical (constructs of the HBM).

For binomial logistic regression to be performed, the following assumptions must be met:

- The dependent variable should be measured on a dichotomous scale
- One or more independent variables which can be either continuous (interval or ratio variable) or categorical (ordinal or nominal)
- Independence of observations and the dependent variable should have mutually exclusive and exhaustive categories^22^.

The procedure gave rise to estimates of odds of a certain event occurring (contraceptive use), given a set of explanatory variables (the constructs of the HBM).

All these assumptions were fulfilled in the current study, thus justifying the use of binomial logistic regression to determine the psychosocial factors influencing contraceptive use among adolescent mothers in the Volta Region of Ghana.

To estimate the odds ratios (ORs), all the components of the HBM and the socio-demographic variables were entered into the first model to evaluate possible interactions. Significant interaction terms were retained and entered in a general model. The sequence of covariate removal from the model was determined by the likelihood ratio testing and the Hosmer-Lemeshow goodness-of-fit test to ensure that the covariate that contributed the least to the fit of the model would be removed first while adjusting for the simultaneous effects of other sets of variables in the model. The significant level for all statistical tests was 5%.

### Ethical consideration

Ethical approval was obtained from the Research Ethics Committee of the University of Health and Allied Sciences UHAS-REC A.5 [5] 18–19. This research was conducted following the accepted principles of ethics in research practice. Administrative approvals were sought from the Regional, Municipal and District health directorate as well as the various health facilities. Written informed consent was obtained from participants on standard consent form before they were included in the study. Confidentiality was fully assured. Due to the sensitive nature of the study, the researcher informed the participants that their names will not be taken so there would not be any situation where a participant's name will be attached to any document of the study. Participants were informed that participation in the study was voluntary and they could decide to withdraw from the study at any time without any consequences.

## Results

### Socio-demographic characteristics of respondents

Table 1 presents the demographic characteristics of adolescent mothers. Of the 442 adolescent mothers surveyed, the mean age was 17.2±1.3 years. The majority (54.3%) of the mothers were below 18 years; (49.3%) had no formal education; (85.6%) were Christians; most (91.2%) were not married and the majority (92.7%) had one child. About 80.1% were Ewes and most (78.9%) were unemployed

**Table 1:** Demographic characteristics of respondents

Characteristics	Frequency (n=422)	Percentage (%)
**Mean age (Range) SD in years**	17.2 (14 – 19) 1.3	
**Age (years)**		
Below 18	229	54.3
18 and above	193	45.7
**Highest level of education**		
None	208	49.3
Basic	197	46.7
Secondary	17	4.0
**Religion**		
Christianity	361	85.6
Islamic	28	6.6
Traditional	33	7.8
**Marital status**		
Married	37	8.8
Not married	385	91.2
**Parity**		
1	391	92.7
More than 1	31	7.3
**Ethnicity**		
Ewe	338	80.1
Guan	29	6.9
Others specify	55	13.0
**Occupation**		
None	333	78.9
Traders	89	21.1

### Contraceptive use among adolescent mothers

Only 18.7% of the adolescent mothers interviewed used contraceptives

### Psychosocial factors influencing contraceptive use based on the Health Belief Model

The psychosocial factors assessed by this study were based on the six constructs of the HBM: perceived susceptibility, perceived severity, perceived benefits, perceived barriers, cues to action and perceived self-efficacy (Table 2).

**Table 2:** Psychosocial factors influencing contraceptive use

Variables	Frequency	Percentage
	N=422	(%)
**Perceived susceptibility**		
***Being susceptible to adolescent pregnancy complications***		
No	329	78.0
Yes	93	22.0
***Contraceptive use can prevent adolescent pregnancy complications***		
No	310	73.5
Yes	112	26.5
**Perceived severity**		
***Pregnancy-related complications can lead to the death of the mother***		
No	275	65.2
Yes	147	34.8
***Pregnancy-related complications can lead to the death of the baby***		
No	310	73.5
Yes	112	26.5
***Pregnancy-related complications can affect the mother's reproductive*** ***system***		
No	317	75.1
Yes	105	24.9
***Pregnancy-related complications can affect a child's brain***		
No	317	75.1
Yes	105	24.9
**Perceived benefits**		
***Contraceptive use prevents pregnancy***		
No	237	56.2
Yes	185	43.8
***Contraceptive use helps in birth spacing***		
No	265	62.8
Yes	157	37.2
***Contraceptive use helps one in achieving personal goals***		
No	248	58.8
Yes	174	41.2
**Perceived barriers**		
***Distance to accessing of Family Planning services***		
No	414	98.1
Yes	8	1.9
***Fear of side effects***		
No	244	57.8
Yes	178	42.2
***Cost of contraceptive***		
No	326	77.3
Yes	96	22.7
***The attitude of service providers***		
No	312	73.9
Yes	110	26.1
***Partner or family opposition***		
No	345	81.8
Yes	77	18.2
**Cues to action**		
***Received information or reminders on contraceptives***		
No	289	68.5
Yes	133	31.5
***Perception of the importance of information or reminders received in*** ***contraceptive use (n=133)***		
No	94	70.7
Yes	39	29.3
**Perceived self-efficacy**		
***Confident to suggest contraceptive use to partner***		
No	341	80.8
Yes	81	19.2
***Confident to ask for contraceptive methods in health facilities***		
No	341	80.8
Yes	81	19.2
***Confident to use contraceptives***		
No	326	77.3
Yes	96	22.7

Concerning perceived susceptibility, the majority (78%) of the mothers indicated they were not susceptible to adolescent pregnancy complications and 73.5% also did not know that contraceptive use can prevent adolescent pregnancy complications. The participants in this study did not perceive adolescent pregnancy to be severe (34.8% to 24.9%) and a slight majority perceived contraceptive use to be beneficial (56.2% to 58.8%). Access to family planning services, fear of side effects, cost of contraceptives, the attitude of service providers and partner opposition were not perceived as barriers to contraceptive use (98.1%, 57.8%, 77.3%, 73.9 and 81.8%) respectively. With regard to cues to action, the majority (68.5%) did not receive any information or reminders on contraceptive use and most (70.7%) did not perceive the information they received to be important. The majority of the mothers were not confident to suggest contraceptive use to their partners, not confident to ask for contraceptive methods in a health facility and not confident to use contraceptives (80.8%, 80.8% & 77.3%) respectively.

### Association between psychosocial factors (constructs of the Health Belief Model) and the odds of contraceptive use (adjusted)

Table 3 shows that after adjusting for confounding variables, adolescent mothers who perceived themselves to be at risk of pregnancy complications were 14 times more likely to use contraceptives as compared to those who did not [OR=13.8(95% CI:3.94–48.05); p<0.001]; those who perceived side-effects of contraceptive as a barrier were 80% less likely to use contraceptives as compared to those who did not [OR=0.2(95% CI:0.05–0.62); p=0.007]; adolescent mothers who were confident to suggest contraceptive use to their partners were 7 times more likely to use contraceptives as compared to those who did not [OR=6.6(95% CI:1.89–23.27); p=0.003]. Also, adolescent mothers who were confident to ask for family planning services at a health facility were 9 times more likely to use contraceptives as compared to those who did not [OR=9.2(95% CI:2.63–32.46); p<0.001]. The adolescent mothers who were confident to use contraceptives were 10 times more likely to use contraceptives as compared to those who were not [OR=9.8(95% CI:2.96–32.16); p<0.001].

**Table 3:** Bivariate and multivariate analysis of factors influencing contraceptive use

Variable	COR (95% CI) p-value	AOR (95% CI) p-value
**Religion**		
Christianity	Ref	Ref
Islamic	2.5 (1.12–5.73) 0.026*	3.9(0.52–29.82) 0.186
Traditional	0.6(0.21–1.85)0.398	0.7(0.09–5.63)0.759
**Parity**		
1	Ref	Ref
More than 1	2.2 (1.00 – 4.93) 0.049*	1.1(0.13–9.50)0.936
**Perceived susceptibility**		
***Adolescent pregnancy complications*** ***without the use of contraceptives***		
No	Ref	Ref
Yes	46.2(23.43–91.08) <0.001*	**13.8(3.94–48.05) <0.001** *
**Perceived severity**		
***Pregnancy-related complications*** ***can lead to the death of the mother***		
No	Ref	Ref
Yes	7.3(4.21–12.59) <0.001*	1.3(0.37–4.94)0.653
**Perceived benefit**		
***Contraceptive use prevents*** ***pregnancy***		
No	Ref	Ref
Yes	7.1(3.95–12.89) <0.001*	2.2(0.30–15.81)0.445
***Contraceptive use helps in birth*** ***spacing***		
No	Ref	Ref
Yes	7.4(4.22–12.89) <0.001*	1.7(0.32–8.80)0.537
***Contraceptive use helps one in*** ***achieving goals (higher education*** ***and a good job)***		
No	Ref	Ref
Yes	8.2(4.55–14.90) <0.001*	0.5(0.06–3.41)0.455
**Perceived barriers**		
***Fear of side effects***		
No	Ref	Ref
Yes	0.3(0.17–0.55) <0.001*	**0.2(0.05–0.62)0.007** *
***The attitude of service providers***		
No	Ref	Ref
Yes	0.4(0.23–0.86)0.017*	0.4(0.10–1.36)0.131
**Cues to action**		
***Received information or reminders*** ***on contraceptive use***		
No	Ref	Ref
Yes	10.7(6.07–18.94) >0.001*	2.8(0.83–9.17)0.098
**Self-efficacy**		
***Confident to suggest use*** ***contraceptive to partner***		
No	Ref	Ref
Yes	48.4(24.56–95.48) <0.001*	**6.6(1.89–23.27)0.003** *
***Confident to ask for family planning*** ***services at a health facility***		
No	Ref	Ref
Yes	38.4(19.97–73.93) <0.001*	**9.2(2.63–32.46) <0.001** *
***Confident to use contraceptives***		
No	Ref	Ref
Yes	24.4(13.24–44.96) <0.001*	**9.8(2.96–32.16) <0.001** *

* p-value significant at 95% CI

## Discussion

The findings from this study showed that the prevalence of contraceptive use among the adolescent mothers was 18.7%. This finding is contrary to the results of a study done in Malawi, which revealed that 45.2% of adolescent mothers reported use of contraceptives^23^. The difference in prevalence between the current study and that of Malawi could be as a result of the high prevalence of HIV in Malawi leading to an increase in contraceptive (condom) use. The low level of contraceptive use in the current study is similar to the results of Nyarko (2015) in Ghana^24^, which revealed that 18.3% of adolescents use contraceptives. The prevalence of contraceptive use observed in the current study is higher compared to that of a study conducted by Mahmood and colleagues in India that reported a prevalence of 13.8%^25^. The difference in prevalence between the current study and that of India could be as a result of the strong religious opposition to contraception by Muslims; thus, the study in India involved more Muslims as compared to the current study. The low prevalence of contraceptive use in the current study calls for health promotion interventions to improve contraceptive use among adolescent mothers in the Volta Region of Ghana.

The current study investigated the psychosocial factors influencing contraceptive use based on the HBM. According to the HBM, an adolescent mother will use contraceptives if she believes she is at risk of adolescent pregnancy complications (perceived susceptibility)^8^,^9^. Adolescent mothers who perceived that they are susceptible to adolescent pregnancy complications (perceived susceptibility) were more likely to use contraceptives compared to those who did not. This finding supports that of a study conducted in Ethiopia, which reported that women who perceived high risk of adolescent pregnancy complications were more likely to use contraceptives as compared to those who did not^26^. The finding also supports that of a study conducted in Malawi^23^, which reported that adolescent mothers who were knowledgeable about adolescent pregnancy complications and susceptible to them were more likely to use contraceptives as compared to those who were not. Furthermore, the finding of the current study is similar to that of a study conducted in Cape Coast, Ghana^27^, in which adolescent mothers who perceived a higher chance of developing adolescent pregnancy complications were found to be more likely to use contraceptives as compared to those who did not. The current finding points to the need for health promotion interventions to heighten the perception of susceptibility to pregnancy complications among adolescents, should they become pregnant. This could motivate them to use contraceptives to prevent these complications by avoiding adolescent pregnancies.

For adolescent mothers to use contraceptives to prevent pregnancy, they have to overcome barriers to contraceptive use. Perceived barriers refer to one's belief in the tangible and psychological costs of the advised behaviours against a condition or problem^8^,^9^. Any obstacle to the use of contraceptives might prevent its usage to prevent pregnancy. Under perceived barriers, 42.2% of the adolescent mothers reported fear of side-effects as a barrier to contraceptive use. Adolescent mothers who perceived side-effects as a barrier to contraceptive use were less likely to use them compared to those who did not. This finding supports that of a study done in Nigeria, which reported that 41.1% of women surveyed reported fear of side-effects as the main reason why they did not use contraceptives^28^ and that of a study done in Democratic Republic of Congo, which found that 31.5% of women stated fear of side-effects as the main barrier to contraceptive use^29^. Also, the result of this current study is compatible with that of a study done in the Northern region of Ghana, which reported that 47.3% of the female adolescents reported adverse side-effect as a reason they did not use contraceptives^30^. The reason for the perception of side-effects of contraceptives reported in the current study could be due to inadequate information, education and communication about contraceptives among the adolescents. There is, therefore, the need for health promotion interventions to deliver accurate knowledge about contraceptives to the adolescent mothers and also strategies to overcome real side-effects to contraceptive use.

It is only when adolescent mothers realise that they can deal with these barriers, that they would be able to use contraceptives. Perceived self-efficacy is the strength of an adolescent mother's belief in her ability to respond to novel or difficult situations and to deal with any associated obstacles or setbacks to contraceptive use^8^,^9^. An adolescent mother should feel that she is capable of using contraceptives because it is that confidence that would motivate her to initiate and sustain the action.

An adolescent mother will use contraceptives to prevent pregnancy complications if she believes that she can successfully use them (perceived self-efficacy). Self-efficacy results showed that adolescent mothers who were confident to ask for contraceptive methods from a health facility were more likely to use contraceptives as compared to those who were not. This finding supports that of a study conducted in Ethiopia^26^, which reported that women who were confident to ask for contraceptive methods from health facilities were more likely to use them as compared to those who were not. Furthermore, adolescent mothers who were confident to suggest to their partners to use contraceptives were more likely to use them as compared to those who were not. This finding agrees with those of a study conducted in Ghana, which reported that women who were confident to suggest to their partners to use contraceptives were more likely to use them as compared to those who were not^31^. Also, adolescent mothers who were confident to use contraceptives were more likely to use them as compared to those who were not. This corroborates the finding of Bader^32^, who reported that adolescent women who were confident to use contraceptives were more likely to use them as compared to those who were not^33^. The findings of self-efficacy in the current study, in general, corroborate that of a study conducted in Uganda, which showed that women in general with low self-efficacy were less likely to use contraceptives as compared to those with high self-efficacy^33^.

The current findings point to the need for health promotion intervention to increase the ability and skills of adolescent mothers to use contraceptives. These interventions could increase the use of contraceptives among adolescent mothers and in turn curb adolescent pregnancy in the Volta Region of Ghana.

The current results should, however, be interpreted in line with some limitations. The convenience sampling used at the last stage of the multistage sampling is a non-probability sampling method and may limit the generalizability of the findings of this research. Also, the study used a cross-sectional design and thus cause-effect relationships could not be ascertained. Also, the study used a self-reporting instrument (questionnaire) that has the potential of introducing social desirability bias and there was no way to validate what the respondents reported. However, the assurance of anonymity and confidentiality of the responses should have minimized possible misreporting. Despite these limitations, this study provides insight into the psychosocial factors influencing contraceptive use among adolescent mothers in the Volta Region using the HBM.

## Conclusion

The level of contraceptive use among adolescent mothers was 18.7%. The HBM constructs which were associated with contraceptive use were perceived susceptibility, perceived barriers and perceived self-efficacy. Programmes and interventions to increase contraceptive use should focus on heightening the perception of risk of adolescents regarding pregnancy complications, reducing barriers to contraceptive use and increasing the skills in negotiating contraceptive use.

The increase in contraceptive use among adolescent mothers in the Volta Region of Ghana can help to achieve two targets of the Sustainable Development Goal (SDG) 3: reducing the global maternal mortality ratio to less than 70 per 100,000 live births and ending preventable deaths of newborns and children under 5 years of age by the year 2030. It can also help to achieve SDG 5: gender equality and empower all women and girls^7^.

## Data Availability

Data will be made available upon request.
